# Cost-effectiveness of umeclidinium compared with tiotropium and glycopyrronium as monotherapy for chronic obstructive pulmonary disease: a UK perspective

**DOI:** 10.1186/s12962-018-0101-3

**Published:** 2018-05-10

**Authors:** Dhvani Shah, Maurice Driessen, Nancy Risebrough, Timothy Baker, Ian Naya, Andrew Briggs, Afisi S. Ismaila

**Affiliations:** 1 ICON Health Economics, ICON, New York, NY USA; 2 Value Evidence & Outcomes, GSK, Brentford, Middlesex, UK; 3 ICON Health Economics, ICON, Toronto, ON Canada; 4 Respiratory Medical Franchise, GSK, Brentford, Middlesex, UK; 5 Health Economics & Health Technology Assessment Institute of Health & Wellbeing, University of Glasgow, Glasgow, UK; 6 Value Evidence & Outcomes, GSK, 5 Moore Drive, PO Box 13398, Research Triangle Park, NC 27709-3398 USA; 7 Department of Health Research Methods, Evidence and Impact, McMaster University, Hamilton, ON Canada

**Keywords:** Chronic obstructive pulmonary disorder, Cost-effectiveness, Economic evaluation, Long-acting muscarinic antagonist

## Abstract

**Background:**

Cost-effectiveness of once-daily umeclidinium bromide (UMEC) was compared with once-daily tiotropium (TIO) and once-daily glycopyrronium (GLY) in patients with chronic obstructive pulmonary disease (COPD) from a UK National Health Service (NHS) perspective.

**Methods:**

A linked-equation model was implemented to estimate COPD progression, associated healthcare costs, exacerbations rates, life years (LY) and quality-adjusted LY (QALYs). Statistical risk equations for endpoints and resource use were derived from the ECLIPSE and TORCH studies, respectively. Treatment effects [mean (standard error)] at 12 weeks on forced expiratory volume in 1 s and St George’s Respiratory Questionnaire score were obtained from the intention-to-treat populations of two head-to-head studies [GSK study identifiers 201316 (NCT02207829) and 201315 (NCT02236611)] which compared UMEC 62.5 mcg with TIO 18 mcg and UMEC 62.5 mcg with GLY 50 mcg, respectively. Treatment costs reflect UK list prices (2016) and NHS unit costs; UMEC and GLY prices being equal and less than TIO. A lifetime horizon, discounted costs and effects at 3.5% were used. Sensitivity analyses were performed to evaluate the robustness of variations in input parameters and assumptions in the model.

**Results:**

Over a lifetime horizon, UMEC was predicted to increase LYs (+ 0.195; 95% confidence interval [CI]: 0.069, 0.356) and QALYs (+ 0.118; 95% CI: 0.055, 0.191) and reduce the number of annual exacerbations (− 0.053; 95% CI: − 0.171, 0.028) compared with TIO, with incremental cost savings of £460/patient (95% CI: − £645, − £240). Compared with GLY, UMEC increased LYs (+ 0.124; 95% CI: 0.015, 0.281) and QALYs (+ 0.101; 95% CI: 0.043, 0.179) and reduced annual exacerbation (− 0.033; 95% CI: − 0.135, 0.017) at an additional cost of £132/patient (95% CI: £12, £330), resulting in an incremental cost-effectiveness ratio of £1310/QALY (95% CI: £284, £2060). Similar results were observed in alternative time horizons and additional sensitivity analyses.

**Conclusions:**

For treatment of patients with COPD in the UK over a lifetime horizon, treatment with UMEC dominates treatment with TIO, providing both improved health outcomes and cost savings. In comparison with GLY, treatment with UMEC achieved improved health outcomes but was associated with a higher cost.

*Trial registration* 201316, NCT02207829; 201315, NCT02236611

**Electronic supplementary material:**

The online version of this article (10.1186/s12962-018-0101-3) contains supplementary material, which is available to authorized users.

## Background

Chronic obstructive pulmonary disease (COPD) is an irreversible and progressive disease characterised by persistent airflow limitation [1, 2]. COPD presents a substantial burden to global public health [3, 4], representing one of the leading causes of hospitalisations and emergency room (ER) visits [3], as well as a leading cause of death worldwide (approximately 3.2 million deaths were attributed to COPD in 2015) [5]. The disease and associated comorbidities contribute significantly to healthcare costs [3, 4], indeed, the financial burden of COPD inevitably correlates with the severity of the disease, with hospital stays accounting for a substantial proportion of the costs [6–8]. In the UK, direct medical costs associated with COPD have been estimated at £1.9 billion per year [9].

The benefits of appropriate pharmacological therapy in COPD in decreasing symptoms, reducing the need for rescue therapy, decreasing the frequency and severity of exacerbations, and improving health-related quality of life (HRQoL), are well recognised and summarised along with treatment recommendations in the annually updated Global Initiative for Chronic Obstructive Lung Disease (GOLD) strategic document [2]. Currently, the recommendation for pharmacological treatment of most patients with COPD is bronchodilator monotherapy or dual therapy with long-acting muscarinic antagonists (LAMAs) and long-acting β_2_-agonists (LABAs), depending on disease burden and exacerbation history [2]. These recommendations reflect the central role of bronchodilators in treating COPD [10, 11]. In addition, LAMAs have been reported to be more effective than LABAs in reducing the rate of exacerbations and reducing the time to a first moderate or severe exacerbation in patients with moderate-to-very severe COPD [12, 13].

Umeclidinium 62.5 mcg (UMEC) is a once-daily LAMA delivered via a novel dry-powder inhaler (Ellipta, GlaxoSmithKline, Hertfordshire, UK), which was approved for maintenance treatment of COPD in the UK in 2014 [14], and is currently approved in multiple regions worldwide, including the United States [15], the European Union [16] and Japan [17]. Several studies have reported the efficacy of UMEC monotherapy versus placebo in terms of improved lung function and other outcomes in patients with COPD [18–20]. Studies have also compared the efficacy of UMEC with that of the other once-daily dry-powder inhaler LAMAs tiotropium 18 mcg (TIO, HandiHaler, Boehringer Ingelheim Pharmaceuticals, Inc, Ridgefield, USA) [21] and glycopyrronium 50 mcg (GLY, Breezhaler, Novartis, Basel, Switzerland) [22]. These studies reported additional improvements in trough forced expiratory volume in 1 s (FEV_1_) at Day 85 of 53 and 33 mL, respectively, with UMEC versus the comparators in the randomised intent-to-treat (ITT) populations [21, 22]. However, limited data are available reporting the relative cost-effectiveness of UMEC in a clinical setting or compared with other once-daily LAMA alternatives. In this study, we assessed the cost-effectiveness of UMEC monotherapy compared with other once-daily LAMAs in patients with COPD from a UK National Health Service (NHS) perspective.

## Methods

### Objectives

Cost-effectiveness analyses were performed comparing UMEC with TIO and GLY using data from two GSK clinical trials: analysis 1 compared UMEC with TIO using data from GSK Study Number 201316 (NCT02207829); [21] analysis 2 compared UMEC with GLY using data from GSK Study Number 201315 (NCT02236611) [22]. These trials are the only two head-to-head studies of these therapies, and therefore constitute the best available evidence.

### Design of the studies included in the analysis

GSK trials 201316 and 201315 were 12-week, multicentre, randomised, parallel-group studies performed between September 2014 and June 2015 [21, 22]. Eligible patients were ≥ 40 years of age with a diagnosis of COPD, a smoking history of ≥ 10 pack-years, a modified Medical Research Council (mMRC) dyspnoea score of ≥ 2, a FEV_1_/forced vital capacity (FEV_1_/FVC) ratio < 0.70 and a post-salbutamol FEV_1_ ≥ 30% and ≤ 70% of predicted values [21, 22].

Patients were randomised 1:1 to receive either UMEC 62.5 mcg via the Ellipta inhaler, or the comparator LAMA [TIO 18 mcg, HandiHaler (NCT02207829); GLY 50 mcg, Breezhaler (NCT02236611)]. The primary endpoint in both trials was non-inferiority in trough FEV_1_ at Day 85 in the per-protocol (PP) populations; other outcomes assessed in the ITT populations of both studies included trough FEV_1_ and trough FVC during the 12-week study period, Transition Dyspnoea Index, St George’s Respiratory Questionnaire (SGRQ) score, COPD Assessment Test score and rescue medication use [21, 22].

UMEC was found to be superior to TIO and non-inferior to GLY in the PP population; results in the ITT population supported these findings (UMEC vs TIO difference: 53 mL, 95% confidence interval [CI]: 25, 81; p < 0.001; UMEC vs GLY difference: 33 mL, 95% CI: 5, 61), while effects on patient-reported outcomes and the incidence of adverse events were similar between UMEC and both comparators [21, 22].

### Cost-effectiveness model

Cost-effectiveness calculations for both analyses 1 and 2 were performed using the GALAXY COPD disease progression model [23]. The model uses a linked risk equation approach to estimate disease progression, associated healthcare costs (e.g. drug costs, cost for hospitalisations and outpatient visits), and impact on quality-adjusted life years (QALYs) and survival. Statistical risk equations for the epidemiological framework and resourcing framework were derived from the Evaluation of COPD Longitudinally to Identify Predictive Surrogate Endpoints (ECLIPSE) [24] and Towards a Revolution in COPD Health (TORCH) [25] studies, respectively. Details of the model, including internal and external validation, have been published previously [23, 26–29]. Disease status measures and resource use were computed annually. No ethics approval was required for the study as data were taken from previously conducted studies that had obtained ethical approval.

### Study perspective

A UK payer perspective was applied [i.e. the UK National Health Service (NHS)] and only direct healthcare costs were considered [30].

### Model inputs

#### Population

All analyses were undertaken using data from the ITT populations in each trial. To ensure that the comparator arms in the model had the same baseline population characteristics, the baseline characteristics were pooled across the treatment arms in each trial.

Data on baseline fibrinogen concentrations and baseline 6-min walk tests (6MWT) were not available from studies 201315 and 201316, and these data were estimated using equations developed within the model using baseline data from the ECLIPSE study [24]; estimated values are presented in Table 1.

**Table 1:** Model inputs: baseline demographics (ITT populations), drug costs and resource costs

Parameters	Analysis 1 (UMEC vs TIO)	Analysis 2 (UMEC vs GLY)
Female, %	28.0	32.0
Age (years), mean (SE)	64.2 (0.3)	64.1 (0.3)
BMI, %		
Low	10.0	10.0
Medium	65.0	61.0
High	25.0	29.0
Any CVD comorbidity, %	64.0	68.0
Any other comorbidity, %	87.0	89.0
No prior exacerbations at baseline, %	69.0	69.0
mMRC score ≥ 2, %	100.0	100.0
Current smokers, %	51.0	47.0
Height (cm), mean (SE)	169.3 (0.3)	168.8 (0.3)
Number of exacerbations in previous year, mean (SE)	0.42 (0.02)	0.40 (0.02)
Number of severe exacerbations, mean	0.13	0.11
Baseline SGRQ score (units), mean (SE)	45.2 (0.6)	44.7 (0.5)
Derived baseline utility, mean	0.730	0.733
Baseline FEV_1_ % predicted, mean (SE)	50.7 (0.3)	50.5 (0.3)
Fibrinogen (mcg/dL), mean (SE)^a^	459.5 (2.37)	461.5 (2.37)
6MWT distance (m)^a^	362.9	361.05
Relative treatment effects (UMEC vs comparator)^b^		
FEV_1_ increment (mL), mean (SE)	53.0 (14.3)	33.0 (14.3)
SGRQ change (units), mean (SE)	− 0.5 (0.8)	− 0.6 (0.8)
Daily drug costs (£)		
UMEC	0.92	0.92
Reference drug	TIO: 1.16	GLY: 0.92
Hospital costs (£)^c^		
ICU (cost/day)	1260	1260
General ward (cost/day)	402	402
COPD-related hospitalisation (cost per episode)	1420	1420
ER visit (cost per visit)	187	187
Outpatient visit (initial visit)	199	199
Outpatient visit (subsequent visit)	147	147
Physician visit costs (£)^d^		
Day time home visit	128	128
Night time home visit	128 (assumption)	128 (assumption)
Visit to physician’s office	65	65
Telephone consultation	27	27

Baseline demographics for each treatment arm were pooled in each analysis. Cost data are presented to three significant figures

*6MWT* 6-min walk test, *BMI* body mass index, *COPD* chronic obstructive pulmonary disease, *CVD* cardiovascular disease, *ER* emergency room, *FEV*_*1*_ forced expiratory volume in 1 s, *GLY* glycopyrronium, *ICU* intensive care unit, *ITT* intent-to-treat, *Mmrc* modified Medical Research Council, *NHS* National Health Service, *SE* standard error, *SGRQ* St George’s Respiratory Questionnaire, *TIO* tiotropium, *UMEC* umeclidinium

^a^ Baseline fibrinogen and 6MWT distance were not available in the trial data but were predicted based on risk equations

^b^ To avoid double-counting of treatment effects an iterative approach was used to adjust the magnitude of the SGRQ treatment effect entered into the model. This ensured that the predicted clinical outcomes matched the observed trial data

^c^ Department of Health, NHS Reference costs 2014–2015 [33]

^d^ Personal Social Services Research Unit. Unit costs of health & social care [32]

#### Efficacy input parameters

Two measures of treatment efficacy were included in the model: between-treatment difference in change from baseline in post-bronchodilator FEV_1_ at 12 weeks and change from baseline in SGRQ score at 12 weeks. The values of the model inputs for these efficacy measures are presented in Table 1. To avoid double counting of treatment effects (i.e. an overlap of SGRQ impact attributable to FEV_1_ improvement and SGRQ impact obtained from the clinical trial), input value for SGRQ treatment effect was calculated as the difference between SGRQ obtained from the clinical trial and SGRQ obtained from the model attributable to FEV_1_ improvement. This ensured that the predicted clinical outcomes matched the observed trial data.

#### Cost input

All costs were based on 2015 Great British Pounds, except for drug costs which were based on price lists accessed in 2016. Costs of treatment with UMEC (Incruse Ellipta, £27.50 per pack), GLY (Seebri Breezehaler, £27.50 per pack) and TIO (Spiriva HandiHaler, £34.87 per pack) were obtained from the Monthly Index of Medical Specialities database [31] (Additional file 1: Table S1) and were estimated for a 30-day supply using dose, pack-size and cost per pack. The model inputs for drug costs and resource costs are presented in Table 1.

Physician fees and hospital costs were obtained from the 2015 Personal Social Services Research Unit [32] or the 2014/2015 NHS National Schedule of Reference Costs [33]. Resource use included annual count predictions for disease-related general ward days, intensive care unit (ICU) days, office visits, day/night home visits, ER visits and outpatient visits. These were based on cohort baseline parameters and concurrent clinical parameters such as exacerbations rates and FEV_1_ increment. Using the risk equation methodology, the predicted resource utilisation counts were multiplied by the relevant unit cost for each year of the model. Hospitalisation costs were assessed using an estimation of ICU and ward day counts. Medication cost index data as of 2016 were obtained from the Monthly Index of Medical Specialities database [34] (Additional file 1: Table S1) and used to estimate daily drug costs (Table 1).

#### Utilities

In each model iteration, SGRQ scores were translated into a EuroQol five dimensions questionnaire (EQ-5D 3L) annual utility estimate using the following relationship [35]:

$$ EQ-5D \,3L= 0.9617-(0.0013*SGRQ\,total) -(0.0001*[SGRQ \, total]^2)+(0.0231*male).$$

#### Model assumptions

Treatment was assumed to continue throughout the modelled lifetime time horizon; it was assumed there was no waning of treatment effect over time, and no treatment discontinuation was included in the analysis. Treatment effects were assumed to start at 0 months.

### Base case settings

A lifetime time horizon was employed as base case due to the chronic and progressive nature of COPD and in accordance with UK guidelines [30]. Brand costs were used for TIO due to the low use of generic TIO in the UK (IMS Health, data on file). Costs and benefits were discounted at 3.5% per annum (p.a.) as base case, in accordance with the National Institute for Health and Care Excellence (NICE) recommendations [30].

### Model outputs

The model estimated: the number of moderate and severe exacerbations over time; the exacerbation rate per patient per year; total, drug-related and non-drug-related costs; the number of life years (LYs) gained (undiscounted); the number of QALYs (discounted) gained and associated healthcare costs (incremental costs/LY gained, incremental cost/QALY gained).

### Sensitivity and scenario analyses

Scenario analyses were conducted for more limited time horizons of 5 and 10 years, discount rates of 0% and 5% p.a., and 1- and 3-year durations of treatment. In addition, scenario analyses were performed for the upper and lower 95% confidence limits of FEV_1_ increment, SGRQ score increment, baseline fibrinogen concentration and baseline 6MWT distance, in order to determine the impact of these variables on the model results. Scenario analyses were also conducted on the price for TIO and a threshold analysis was run to determine the price of TIO which would lead to the same overall costs for both arms in the analysis.

### Probabilistic sensitivity analysis

A probabilistic sensitivity analysis (PSA) was conducted to test the robustness of the results derived from the base case analysis and to evaluate the impact of uncertainty in the parameters used within the model. This was conducted by assigning distributions to input parameters and randomly sampling from these distributions over 1000 Monte Carlo simulations. A normal distribution was used for treatment effects and risk equations were derived using correlated draws from a Cholesky decomposition table obtained from the covariance matrices for each equation. The outputs of this analysis were summarised in scatter plots of incremental costs and effectiveness, and net benefit acceptability curves (NBAC) for competing treatments included in the model.

## Results

### Comparison of UMEC with TIO

In the lifetime analysis, estimated total accumulated costs were £12,300 and £12,800 for UMEC and TIO respectively (Table 2). UMEC provided an additional 0.195 LYs and 0.118 QALYs compared with TIO, with cost savings of £460 (Table 2). Patients on UMEC also had fewer exacerbations per patient per year (0.718) compared with TIO (0.740) (Table 2). Despite higher healthcare costs with UMEC, reflecting increased survival times, there were overall cost savings due to the lower drug costs of UMEC compared with TIO. UMEC therefore dominated TIO, providing both improved outcomes and cost savings.

**Table 2:** Model results: lifetime horizon

	Analysis 1 (UMEC vs TIO)		Analysis 2 (UMEC vs GLY)	
	TIO	UMEC	GLY	UMEC
Cumulative number of exacerbations				
Moderate	4.926	4.924	4.952	4.951
Severe	1.659	1.608	1.636	1.604
TOTAL	6.585	6.532	6.588	6.555
Severe exacerbations PPPY	0.186	0.177	0.184	0.178
Total exacerbations PPPY	0.740	0.718	0.740	0.726
Outcomes				
Accumulated LYs (undiscounted)	8.902	9.097	8.906	9.029
Accumulated QALYs	5.003	5.121	5.038	5.139
Costs				
Accumulated costs (total)	£12,800	£12,300	£11,800	£12,000
Drug costs	£3180	£2560	£2510	£2540
Non-drug costs	£9590	£9760	£9310	£9420
Hospital costs	£8800	£8960	£8550	£8640
Outpatient/hospital/clinic costs	£504	£514	£480	£486
Physician visits (office, home, day or night)	£288	£292	£288	£290
Incremental results (95% CI), UMEC vs comparator				
Incremental exacerbations		− 0.053 (− 0.171, 0.028)		− 0.033 (− 0.135, 0.017)
Incremental cost		− £460 (− £645, − £240)		£132 (£12, £330)
Incremental LYs		0.195 (0.069, 0.356)		0.124 (0.015, 0.281)
Incremental QALYs		0.118 (0.055, 0.191)		0.101 (0.043, 0.179)
ICER (QALY)		Dominant		£1310 (£284, £2060)
ICER (LY)		Dominant		£1070 (£718, £1520)

Cost and cost-effectiveness data are presented to three significant figures for values of three figures or more, and to the nearest pound for values rounding to less than 100

*CI* confidence interval, *GLY* glycopyrronium, *ICER* incremental cost-effectiveness ratio, *LY* life year, *PPPY* per person per year, *QALY* quality-adjusted life year, *TIO* tiotropium, *UMEC* umeclidinium

Results were similar in the analyses with 5- and 10-year horizons, with UMEC showing improved outcomes (0.018 and 0.082 LYs; 0.029 and 0.072 QALYs, respectively) and cost savings compared with TIO (£355 and £486, respectively) (Additional file 1: Tables S2 and S3).

**Fig. 1:**
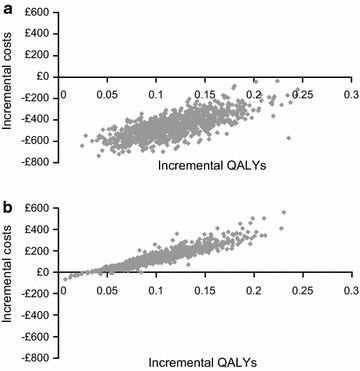
Cost-effectiveness scatter plots for **a** UMEC versus TIO and **b** UMEC versus GLY, lifetime horizon. *GL*, glycopyrronium, *QALY* quality-adjusted life year, *TIO* tiotropium, *UMEC* umeclidinium

In the PSA, UMEC was less costly than TIO, and achieved a greater gain in QALYS than TIO, in 100% of the simulations in the cost-effectiveness scatter plot (Fig. 1a). The NBAC (Additional file 1: Figure S1A) showed that UMEC generated higher net monetary benefit in all simulations.

In the scenario analyses, UMEC continued to dominate TIO across most scenarios (Additional file 1: Table S4). However, in three scenarios, UMEC did not dominate TIO: the scenario in which SGRQ upper confidence limit was tested [incremental cost-effectiveness ratio (ICER) = £64,500/QALY], the scenario using generic costs for TIO (£25.80 per pack, ICER = £3130/QALY), and the scenario in which the cost of TIO was assumed to be same as UMEC (ICER = £1810/QALY). Threshold analyses were performed to determine the price for TIO which would lead to the same overall costs for both arms in the analyses. This price was identified as £29.83 for 30 inhalations, therefore if TIO was priced lower than this value, UMEC would no longer be dominant.

### Comparison of UMEC with GLY

In the lifetime analysis, estimated total accumulated costs were £12,000 and £11,800 for UMEC and GLY respectively (Table 2). Against similar drug costs, UMEC provided an additional 0.124 LYs and 0.101 QALYs compared with GLY, with an additional incremental cost of £132 (Table 2). The estimated ICER was £1070/LY and £1310/QALY gained. Patients on UMEC also had fewer exacerbations per patient per year (0.726) compared with GLY (0.740) (Table 2). However, all costs were higher with UMEC compared with GLY.

Results were similar in the analyses with 5- and 10-year horizons, with UMEC showing improved outcomes (0.012 and 0.052 LYs; 0.032 and 0.068 QALYs, respectively) and higher costs (£14 and £61 respectively) compared with GLY (Additional file 1: Tables S2 and S3).

Results of the PSA showed that out of 1000 simulations, 95% resulted in an ICER/QALY of £284–£2060 (Fig. 1b). The NBAC (Additional file 1: Figure S1B) showed that UMEC generated higher net monetary benefit in all simulations above a willingness-to-pay threshold of £5000 per additional QALY.

In the scenario analyses, results remained consistent with the base case analysis (Additional file 1: Table S4), except for the SGRQ upper confidence limit: in this scenario, the reduction in SGRQ for UMEC was lower than for GLY, hence this scenario resulted in UMEC being dominated by GLY. ICERs for other scenarios ranged from £113/QALY (duration of treatment = 1 year) to £1670/QALY (FEV_1_ upper confidence limit). The estimated ICERs were £1430/QALY and £1260/QALY for costs and benefit discount rates of 0% and 5% p.a., respectively. The analyses were most sensitive to time horizon, duration of treatment effect, change in SGRQ score and FEV_1_ increment.

## Discussion

This study showed that UMEC provided gains in LYs and QALYs, and numerical reductions in moderate and severe exacerbation rates, compared with both TIO and GLY. The lifetime analysis demonstrated that UMEC dominated TIO, providing improved outcomes with reduced treatment and resource costs. This dominance was maintained when a 5- and 10-year horizon was employed, and across several scenario analyses. However, when price parity was applied, UMEC no longer dominated TIO, with an ICER of £1810/QALY. Results of the scenario analyses showed the model was most sensitive to scenarios in which the duration of treatment was 1 or 3 years, and SGRQ change, while the model was relatively insensitive to discount rate and upper confidence limits for FEV_1_ increment, 6MWT and fibrinogen data. In comparison with GLY, results obtained from the lifetime horizon showed that costs associated with UMEC were higher. This could be attributed to the increased drug costs, as well as to the survival benefit associated with UMEC, as patients remained in the model for longer and therefore incurred increased treatment and resource costs. These results were generally consistent across other time horizons and scenario analyses. Nevertheless, UMEC was shown to be cost-effective compared with GLY in most scenarios, with cost-effectiveness falling well below the willingness-to-pay threshold of £20,000–30,000 per QALY set by the NICE guidelines [36].

Data comparing cost-effectiveness of different LAMA monotherapies are scarce. One study compared the cost-effectiveness of TIO with GLY in Sweden [37], and one study compared the cost-effectiveness of these treatment in Canada, Spain, Sweden and the UK [38, 39]. In the former study, GLY was found to be less costly and more effective than TIO as maintenance treatment for patients with moderate to severe COPD in Sweden [37]. In the latter study, TIO generated improved outcomes compared with GLY in all countries, and was cost-saving compared with GLY in Canada and the UK. Costs per QALY were positive for Spain and Sweden; however, the estimated ICERs remained below the respective willingness-to-pay thresholds for each country [38]. The outcomes from this latter study contrast with the results of the current cost-effectiveness analysis. Possible reasons for this could include the use of different efficacy parameters, such as the assessment of treatment efficacy including exacerbation rates in the multi-country study. It is also likely that the relative costs of the different drugs in each country would drive the differences in the cost-effectiveness. Additionally, the multi-country study included data from patients with very severe COPD, while this study excluded such patients who would not be ideal candidates for LAMA monotherapy in accordance with the GOLD 2017 COPD strategy report [40, 41]. An additional advantage of the model used in the current study was that it could integrate multiple factors affecting outcomes in COPD to predict disease progression and economic outcomes [23]. This may enable more accurate predictions than other models commonly used in cost-effectiveness analyses that are based only upon disease progression, with disease severity classified according to measurements of percentage predicted FEV_1_ [42].

The efficacy inputs used for this study are based on bronchodilation, as measured by FEV_1_ [21, 22]. Improvements in lung function measured by increased FEV_1_ can result in better health outcomes and lower COPD exacerbation risk over a longer period [21, 22, 43], and may be responsible for the larger treatment effect observed on the cumulative number of severe versus moderate exacerbations. However, the use of a LAMA as monotherapy is not common, as a large proportion of patients with COPD will step-up to dual bronchodilator therapy with a LAMA/LABA or the combination of a LAMA with inhaled corticosteroid (ICS)/LABA. Studies have shown greater improvements in trough FEV_1_ when using a dual LABA/LAMA therapy compared to LAMA monotherapy. A recent systematic review and meta-analysis compared the efficacy and safety of LABA/LAMA with LAMA or ICS/LABA in adults with moderate to severe COPD and found that the LAMA/LABA combinations provided greater improvements in trough FEV_1_ than LAMA monotherapy [44]. Studies using UMEC in combination with the LABA vilanterol 25 mcg (VI) showed a clinically meaningful and significant improvement in trough FEV_1_ versus TIO [45]. UMEC/VI was also found to be potentially cost-effective compared with TIO, with an ICER of €21,475/QALY [46]. Although triple therapies have shown to improve lung function and quality of life compared to ICS/LABA [47], data on the cost-effectiveness of triple therapy in patients with COPD are lacking.

As the studies included in the model lacked data on baseline fibrinogen concentrations and 6MWT distances, these were estimated using predictive equations within the model. Scenario analyses with variable lower and upper confidence limit values for both variables showed minimal changes in the overall results. Nevertheless, it would be highly recommended to collect these variables in future studies for which analysis may be performed using the current model.

At this moment, there are no trials providing data on the use of LAMA therapy for a longer duration than the scenario analyses ran in the current study (5, 10 years and lifetime). Therefore, we needed to make assumptions in order to run the analyses for these scenarios. One of the assumptions was to maintain treatment effects without waning until the end of the year. This assumption was supported by data from the studies included in the model, demonstrating a rapid response to treatment that was stable over the last 2 months of the assessment periods [21, 22]. In addition, data from the Understanding Potential Long-Term Impacts on Function with Tiotropium (UPLIFT) trial investigating the use of LAMA therapy over a 4-year period did not show a decline in treatment effect over time. Indeed, sustained improvements were observed on several outcomes in addition to lung function, such as HRQoL, risk of exacerbations, exacerbation-related hospitalisations and reductions in respiratory and cardiac associated morbidity [40].

Due to the chronic and progressive nature of COPD, and in accordance with UK guidance for the conduct of economic evaluations [30], a lifetime horizon was employed. No suitable data could be identified to estimate treatment discontinuation or switch rates for the analyses presented. Therefore, another assumption was made within the model: that patients did not discontinue or switch their treatment. In reality, stability of treatment is unlikely, as patients will usually escalate from LAMA monotherapy to combined therapies, especially as their disease progresses, in accordance with treatment recommendations [2]. However, the efficacy of LAMA therapy is not expected to be diminished in these scenarios. Indeed, recently published data indicate that efficacy differences between LAMA therapies are still present when administered as part of dual LAMA/LABA combination therapies [48]. Our approach is therefore a conservative estimate; however, we believe that based on the available data this was the most appropriate approach. In a similar vein, the model was built upon the assumption that treatment continued within each modelled time horizon (i.e. over a lifetime horizon, a 10-year horizon and a 5-year horizon). As such, costs and effects were built upon the assumption of patient adherence over each time horizon. The model is therefore not necessarily built to reflect treatment changes as the disease progresses in each individual patient.

## Conclusions

For treatment of patients with COPD in the UK, over a lifetime horizon, treatment with UMEC dominates treatment with TIO. In comparison with GLY, treatment with UMEC achieved improved outcomes with fewer exacerbations; however, treatment and resource costs were higher due to the higher drug costs and increased survival time. UMEC can therefore be considered a dominant treatment option compared to TIO and a cost-effective treatment option compared to GLY. These data may aid payers in making judgements on which LAMA treatments can be considered cost-effective in a UK setting.

## Additional file

**Additional file 1:** **Table S1.** Model inputs: drug cost. **Table S2.** Model results: 5-year horizon. **Table S3.** Model results: 10-year horizon. **Table S4.** Model results: Scenario Analyses UMEC versus TIO (Analysis 1) and UMEC versus GLY (Analysis 2). **Figure S1.** Net benefit acceptability curves for UMEC versus TIO (A) and UMEC versus GLY (B).

## Abbreviations

6MWT: 6-min walk test
CI: confidence interval
COPD: chronic obstructive pulmonary disease
ECLIPSE: Evaluation of COPD Longitudinally to Identify Predictive Surrogate Endpoints (study)
EQ-5D: EuroQol five dimensions questionnaire
ER: emergency room
FEV_1_: forced expiratory volume in 1 s
FVC: forced vital capacity
GLY: glycopyrronium
GOLD: Global Initiative for Chronic Obstructive Lung Disease
HRQoL: health-related quality of life
ICER: incremental cost-effectiveness ratio
ICS: inhaled corticosteroid
ICU: intensive care unit
ITT: intent-to-treat
LABA: long-acting β_2_-agonist
LAMA: long-acting muscarinic antagonists
LY: life year
mMRC: modified Medical Research Council
NBAC: net benefit acceptability curve
NHS: National Health Service
NICE: National Institute for Health and Care Excellence
PP: per-protocol
PSA: probabilistic sensitivity analysis
QALY: quality-adjusted life year
SGRQ: St. George’s respiratory questionnaire
TIO: tiotropium
TORCH: Towards a Revolution in COPD Health (study)
UK: United Kingdom
UMEC: umeclidinium
VI: vilanterol
