# Supplementary material for: Cost-effectiveness of umeclidinium compared with tiotropium and glycopyrronium as monotherapy for chronic obstructive pulmonary disease: a UK perspective
Source: Cost Eff Resour Alloc. 2018 May 10;16:17. doi: 10.1186/s12962-018-0101-3 (PMC5946544; doi:10.1186/s12962-018-0101-3)
Supplement: Supplementary file 1 — Additional file 1: Table S1. Model inputs: drug cost. Table S2. Model results: 5-year horizon. Table S3. Model results: 10-year horizon. Table S4. Model results: Scenario Analyses UMEC versus TIO (Analysis 1) and UMEC versus GLY (Analysis 2). Figure S1. Net benefit acceptability curves for UMEC versus TIO (A) and UMEC versus GLY (B). [file 12962_2018_101_MOESM1_ESM.docx]

# ADDITIONAL FILES

**Additional Figure 1: Net benefit acceptability curves for UMEC versus TIO (A) and UMEC versus GLY (B)**


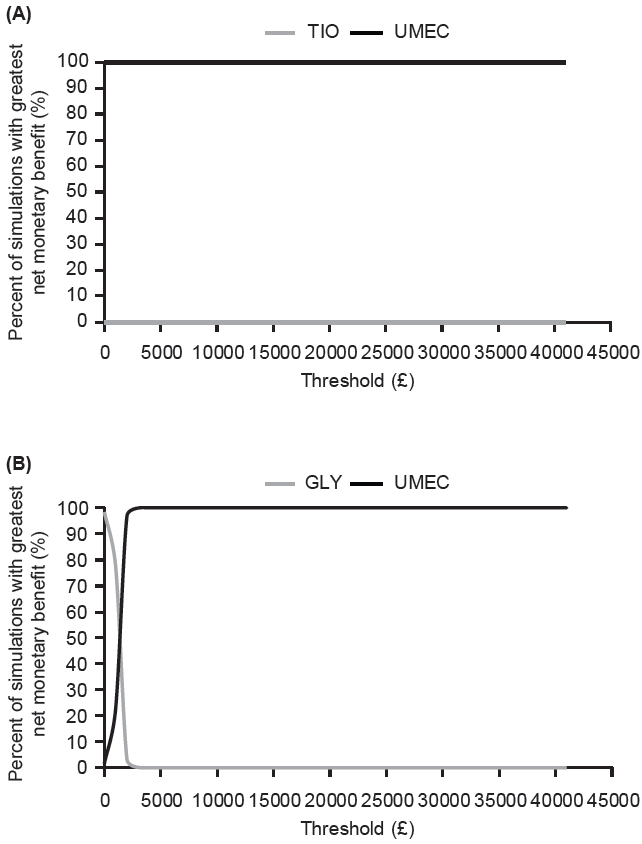


GLY, glycopyrronium; TIO, tiotropium; UMEC, umeclidinium.

**Additional Table 1. Model inputs: drug cost**

| **Drug** | **Dose** | **Pack size** | **Pack cost** | **Cost per dose** | **Cost per day** | **Label dosing** |
| --- | --- | --- | --- | --- | --- | --- |
| Umeclidinium (Incruse Ellipta) | 55 µg | 30 | £27.50 | £0.92 | £0.92 | 1 actuations per day |
| Tiotropium (Spiriva HandiHaler) | 18 µg | 30 | £34.87 | £1.16 | £1.16 | 1 daily by inhalation |
| Tiotropium (Braltus*) | 13 µg | 30 | £25.80 | £0.86 | £0.86 | 1 daily by inhalation |
| Glycopyrronium (Seebri Breezehaler) | 50 µg | 30 | £27.50 | £0.92 | £0.92 | 1 daily by inhalation |
| *Braltus is a generic product; Source: Monthly Index of Medical Specialities. Available at <http://www.mims.co.uk/>, Accessed September 2016. | | | | | | |

**Additional Table 2: Model results: 5-year horizon**

|  | **Analysis 1**  **(UMEC vs TIO)** | | | | **Analysis 2**  **(UMEC vs GLY)** | | |
| --- | --- | --- | --- | --- | --- | --- | --- |
|  | **TIO** | | **UMEC** | **GLY** | | | **UMEC** |
| Cumulative number of exacerbations |  |  | | |  |  | |
| Moderate | 2.485 | | 2.441 | 2.500 | | | 2.472 |
| Severe | 0.593 | | 0.561 | 0.588 | | | 0.568 |
| TOTAL | 3.078 | | 3.002 | 3.088 | | | 3.040 |
| Severe exacerbations PPPY | 0.132 | | 0.124 | 0.131 | | | 0.126 |
| Total exacerbations PPPY | 0.685 | | 0.666 | 0.688 | | | 0.676 |
| Outcomes |  | |  |  | | |  |
| Accumulated LYs (undiscounted) | 4.492 | | 4.511 | 4.488 | | | 4.500 |
| Accumulated QALYs | 2.944 | | 2.974 | 2.960 | | | 2.992 |
| Costs |  | |  |  | | |  |
| Accumulated costs (total) | £6950 | | £6590 | £6410 | | | £6430 |
| Drug costs | £1790 | | £1420 | £1410 | | | £1410 |
| Non-drug costs | £5160 | | £5180 | £5010 | | | £5020 |
| Hospital costs | £4710 | | £4720 | £4570 | | | £4580 |
| Outpatient/hospital/clinic costs | £296 | | £298 | £282 | | | £283 |
| Physician visits (office, home, day or night) | £158 | | £158 | £158 | | | £158 |
| Incremental results (95% CI), UMEC vs comparator | | | | | | | |
| Incremental exacerbations | -0.076 (-0.117, -0.034) | | | | -0.048 (-0.091, -0.007) | | |
| Incremental cost | -£355  (-£381, -£313) | | | | £14  (£0, £40) | | |
| Incremental LYs | 0.018  (0.006, 0.036) | | | | 0.012  (0.001, 0.027) | | |
| Incremental QALYs | 0.029  (0.015, 0.044) | | | | 0.032  (0.019, 0.047) | | |
| ICER (QALY) | Dominant | | | | £440  (£0, £932) | | |
| ICER (LY) | Dominant | | | | £1200  (£339, £2300) | | |

Cost and cost-effectiveness data are presented to three significant figures for values of three figures or more, and to the nearest pound for values rounding to less than 100.

CI, confidence interval; GLY, glycopyrronium; ICER, incremental cost-effectiveness ratio; LY, life-year; PPPY, per person per year; QALY, quality-adjusted life-year; TIO, tiotropium; UMEC, umeclidinium.

**Additional Table 3: Model results: 10-year horizon**

|  | **Analysis 1**  **(UMEC vs TIO)** | | **Analysis 2**  **(UMEC vs GLY)** | | |
| --- | --- | --- | --- | --- | --- |
|  | **TIO** | **UMEC** | | **GLY** | **UMEC** |
| Cumulative number of exacerbations |  |  | |  |  |
| Moderate | 4.063 | 4.017 | | 4.082 | 4.053 |
| Severe | 1.190 | 1.134 | | 1.175 | 1.140 |
| TOTAL | 5.253 | 5.152 | | 5.256 | 5.193 |
| Severe exacerbations PPPY | 0.161 | 0.152 | | 0.159 | 0.153 |
| Total exacerbations PPPY | 0.710 | 0.689 | | 0.711 | 0.698 |
| Outcomes |  |  | |  |  |
| Accumulated LYs (undiscounted) | 7.397 | 7.479 | | 7.388 | 7.440 |
| Accumulated QALYs | 4.433 | 4.505 | | 4.458 | 4.526 |
| Costs |  |  | |  |  |
| Accumulated costs (total) | £11,000 | £10,500 | | £10,100 | £10,210 |
| Drug costs | £2770 | £2200 | | £2180 | £2190 |
| Non-drug costs | £8210 | £8290 | | £7970 | £8010 |
| Hospital costs | £7520 | £7590 | | £7300 | £7340 |
| Outpatient/hospital/clinic costs | £444 | £449 | | £423 | £426 |
| Physician visits (office, home, day or night) | £248 | £249 | | £248 | £248 |
| Incremental results (95% CI), UMEC vs comparator | | | | | |
| Incremental exacerbations | -0.101 (-0.184, -0.038) | | -0.064 (-0.144, -0.009) | | |
| Incremental cost | £-486  (-£581, -£333) | | £61  (£5, £151) | | |
| Incremental LYs | 0.082  (0.027, 0.165) | | 0.052  (0.007, 0.117) | | |
| Incremental QALYs | 0.072  (0.035, 0.124) | | 0.068  (0.034, 0.110) | | |
| ICER (QALY) | Dominant | | £898  (£139, £1530) | | |
| ICER (LY) | Dominant | | £1170  (£648, £1850) | | |

Cost and cost-effectiveness data are presented to three significant figures for values of three figures or more, and to the nearest pound for values rounding to less than 100.

CI, confidence interval; GLY, glycopyrronium; ICER, incremental cost-effectiveness ratio; LY, life year; PPPY, per person per year; QALY, quality-adjusted life year; TIO, tiotropium; UMEC, umeclidinium.

**Additional Table 4: Model results: Scenario Analyses UMEC versus TIO (Analysis 1) and UMEC versus GLY (Analysis 2)**

|  | **Incremental costs** | | **Incremental QALYs** | | **ICER** | | |
| --- | --- | --- | --- | --- | --- | --- | --- |
| **Scenario** | **Analysis 1** | **Analysis 2** | **Analysis 1** | **Analysis 2** | **Analysis 1** | **Analysis 2** |  |
| Time horizon – 1 year | -£91 | -£1 | 0.003 | 0.005 | Dominant | Dominant | |
| Discount rate (costs and benefits) 0% p.a. | -£493 | £189 | 0.158 | 0.132 | Dominant | £1430 | |
| Discount rate (costs and benefits) 5% p.a. | -£446 | £114 | 0.105 | 0.091 | Dominant | £1260 | |
| Duration of treatment = 1 year | -£672 | £1 | 0.004 | 0.007 | Dominant | £113 | |
| Duration of treatment = 3 years | -£665 | £5 | 0.015 | 0.020 | Dominant | £253 | |
| FEV_1_ UCL | -£346 | £246 | 0.161 | 0.147 | Dominant | £1670 | |
| FEV_1_ LCL | -£573 | £20 | 0.074 | 0.056 | Dominant | £354 | |
| Fibrinogen UCL | -£457 | £132 | 0.117 | 0.101 | Dominant | £1310 | |
| Fibrinogen LCL | -£463 | £133 | 0.118 | 0.101 | Dominant | £1310 | |
| 6MWT UCL | -£459 | £144 | 0.126 | 0.107 | Dominant | £1340 | |
| 6MWT LCL | -£458 | £142 | 0.124 | 0.106 | Dominant | £1340 | |
| SGRQ UCL | -£460 | £132 | -0.007 | -0.016 | £64,500 | Dominated | |
| SGRQ LCL | -£460 | £132 | 0.238 | 0.216 | Dominant | £612 | |
| Generic costs for TIO (Braltus) | £368 | N/A | 0.118 | N/A | £3130 | N/A | |
| TIO price same as UMEC | £213 | N/A | 0.118 | N/A | £1810 | N/A | |

Cost and cost-effectiveness data are presented to three significant figures for values of three figures or more, and to the nearest pound for values rounding to less than 100. UCL and LCL for Analysis 1 (UMEC vs TIO): FEV_1_, 81 mL and 25 mL, respectively; fibrinogen, 464.1 mcg/dL and 454.9 mcg/dL, respectively; 6MWT, 368.3 m and 357.6 m, respectively; SGRQ, 1.13 units and ‑2.04 units, respectively.UCL and LCL for Analysis 2 (UMEC vs GLY): FEV_1_, 61 mL and 5 mL, respectively; fibrinogen, 466.2 mcg/dL and 456.9 mcg/dL, respectively; 6MWT, 366.4 m and 355.7 m, respectively; SGRQ, 0.88 units and -2.17 units, respectively. Generic cost for TIO: £0.86 per day. TIO same price as UMEC: £0.92 per day.

FEV_1_, forced expiratory volume in 1 second; GLY, glycopyrronium; ICER, incremental cost-effectiveness ratio; LCL; lower confidence limit; p.a., per annum; QALY, quality-adjusted life year; SGRQ, Saint George’s Respiratory Questionnaire; TIO, tiotropium; UCL, upper confidence limit; UMEC, umeclidinium; 6MWT, 6-minute walk test.
